# Inflammatory discordance phenotype in COPD: Dissociation between neutrophil-to-lymphocyte ratio and conventional markers

**DOI:** 10.1371/journal.pone.0357923

**Published:** 2026-09-11

**Authors:** Can Colakoglu, Sefa Levent Ozsahin

**Affiliations:** Department of Pulmonary Medicine, Sivas Cumhuriyet University Faculty of Medicine, Sivas, Türkiye; Azienda Ospedaliero Universitaria Careggi, ITALY

## Abstract

**Background:**

Chronic obstructive pulmonary disease (COPD) is a heterogeneous disorder with diverse inflammatory profiles. Long-term oxygen therapy (LTOT) identifies a subgroup of patients with advanced disease; however, the systemic inflammatory characteristics of this population remain incompletely defined.

**Objective:**

To investigate systemic inflammatory markers in clinically stable COPD patients according to LTOT status and to examine whether elevated NLR reflects conventional inflammatory activation or a distinct immune profile.

**Methods:**

This study represents a secondary, exploratory subgroup analysis of a prospectively collected COPD cohort. Patients were stratified according to LTOT status and compared in terms of pulmonary function, gas exchange parameters, and inflammatory markers. NLR was analyzed in relation to total leukocyte count (WBC), C-reactive protein (CRP), eosinophil count, and oxygenation parameters. Between-group comparisons were performed using non-parametric tests, and correlation and multivariable regression analyses were conducted to identify independent associations with NLR.

**Results:**

A total of 127 clinically stable COPD patients were included, of whom 31 (24.4%) were receiving LTOT. LTOT-treated patients had significantly higher NLR values compared with non-LTOT patients (6.32 ± 4.87 vs 3.68 ± 2.41, p = 0.001), accompanied by lower lymphocyte and eosinophil counts. In contrast, total leukocyte count and CRP levels were similar between groups. In multivariable analysis, WBC, eosinophil count, and CRP remained independently associated with NLR, whereas oxygenation parameters and pulmonary function measures were not.

**Conclusions:**

This study suggests that NLR elevation in advanced COPD may not necessarily reflect overt systemic inflammation, but rather a distinct immune phenotype. Context-specific interpretation of NLR may therefore be required in advanced disease.

## Introduction

Chronic obstructive pulmonary disease (COPD) is a complex and heterogeneous disorder characterized by persistent airflow limitation and diverse clinical manifestations extending beyond the respiratory system [1]. The recognition of COPD as a systemic disease has led to increased interest in understanding distinct phenotypes that may differ in their inflammatory profiles, clinical course, and therapeutic responses [2,3]. Among these phenotypes, patients with advanced disease requiring long-term oxygen therapy (LTOT) represent a particularly vulnerable subgroup characterized by chronic hypoxemic respiratory failure, frequent exacerbations, and poor prognosis [4,5].

Systemic inflammation has been established as a key component of COPD pathophysiology, contributing to disease progression, exacerbation frequency, and extrapulmonary manifestations [6,7]. Traditional inflammatory markers such as C-reactive protein (CRP) and total white blood cell count (WBC) have been widely used to assess systemic inflammatory burden in COPD patients. However, these conventional markers may not fully capture the complexity of immune responses across different disease phenotypes [8].

The neutrophil-to-lymphocyte ratio (NLR) has emerged as a simple, cost-effective biomarker that reflects the balance between innate and adaptive immune responses [9]. In COPD, elevated NLR has been associated with disease severity, exacerbation risk, and mortality [10–13]. However, an important question remains regarding the biological significance of NLR elevation in different clinical contexts. Specifically, it is unclear whether increased NLR consistently reflects classical systemic inflammatory activation or may represent alternative immune mechanisms, particularly in advanced disease states.

In patients with chronic hypoxemia requiring LTOT, multiple factors including hypoxic stress, neurohumoral activation, and metabolic alterations may influence immune cell distribution and function [14,15]. These mechanisms could potentially lead to changes in circulating immune cell populations without necessarily triggering the acute-phase response typically associated with systemic inflammation [16]. This possibility suggests that NLR elevation in advanced COPD might represent a distinct immunological phenotype rather than simply reflecting increased inflammatory burden.

Understanding the relationship between NLR and conventional inflammatory markers in advanced COPD has important clinical implications. If NLR elevation occurs independent of classical inflammatory activation, this would suggest that therapeutic strategies targeting systemic inflammation might be less effective in this patient population, and alternative approaches addressing specific immune dysregulation might be required.

Therefore, the aim of this study was to investigate the inflammatory profile of clinically stable COPD patients with chronic hypoxemia, as defined by LTOT status, with particular focus on the relationship between NLR and conventional inflammatory markers. We hypothesized that patients requiring LTOT would exhibit elevated NLR values without corresponding increases in traditional inflammatory indicators, suggesting a dissociation between NLR and classical systemic inflammatory activation in advanced disease.

## Materials and methods

### Study design and setting

This study represents a secondary, exploratory analysis of a prospectively collected dataset from the Department of Pulmonology, Cumhuriyet University Faculty of Medicine. Clinical, laboratory, and functional data were originally obtained as part of a comprehensive evaluation of systemic inflammatory markers in patients with chronic obstructive pulmonary disease (COPD). The original data were prospectively collected between 01 January 2017–31 December 2017.

For the present secondary analysis, data were retrospectively accessed for research purposes between 01 October 2025–31 December 2025. The dataset was fully anonymized prior to analysis, and the authors had no access to information that could identify individual participants during or after data collection.

These prospectively collected data were retrospectively reviewed to investigate inflammatory profiles according to long-term oxygen therapy (LTOT) status. Patients receiving LTOT were considered to represent a subgroup with chronic hypoxemic respiratory failure and advanced disease, and were compared with those not requiring oxygen supplementation.

The study was conducted in accordance with the Declaration of Helsinki and approved by the Cumhuriyet University Clinical Research Ethics Committee (Decision No: 2016-12/19). Written informed consent was obtained from all participants at the time of original data collection. The study is reported in accordance with the Strengthening the Reporting of Observational Studies in Epidemiology (STROBE) statement.

### Study population

#### Inclusion criteria.

Patients were eligible for inclusion if they met the following criteria:

1. Confirmed diagnosis of COPD based on post-bronchodilator spirometry (FEV_1_/FVC < 0.70) according to Global Initiative for Chronic Obstructive Lung Disease (GOLD) criteria
2. Clinically stable disease at the time of evaluation (defined as no exacerbation requiring antibiotics, systemic corticosteroids, or hospitalization within the preceding 4 weeks)
3. Complete clinical, laboratory, and pulmonary function data available
4. Age ≥ 40 years

#### Exclusion criteria.

Patients were excluded if they had any of the following:

1. Acute or recent COPD exacerbation (within 4 weeks)
2. Coexisting respiratory diseases (asthma, interstitial lung disease, bronchiectasis, lung cancer)
3. Active hematologic malignancies or immunosuppressive therapy
4. Autoimmune diseases or chronic inflammatory conditions
5. Active infectious diseases or fever at the time of evaluation
6. Recent surgery or trauma (within 4 weeks)
7. Pregnancy
8. Incomplete clinical or laboratory data

### Definition of long-term oxygen therapy

Patients were classified as LTOT-positive if they were receiving prescribed domiciliary oxygen therapy for chronic hypoxemia according to established clinical guidelines at the time of evaluation. LTOT prescription was based on standard criteria including PaO_2_ ≤ 55 mmHg or PaO_2_ 55–60 mmHg with evidence of cor pulmonale or polycythemia.

### Data collection

#### Clinical and demographic variables.

Demographic characteristics including age, sex, body mass index (BMI), smoking history (pack-years), and current smoking status were recorded using standardized case report forms. Duration of bronchodilator therapy and presence of comorbidities were also documented.

#### Pulmonary function testing.

Spirometry was performed using a standardized system (MasterScreen PFT, CareFusion, Germany) according to American Thoracic Society/European Respiratory Society guidelines. Post-bronchodilator measurements included forced expiratory volume in one second (FEV_1_), forced vital capacity (FVC), and FEV_1_/FVC ratio, recorded as both absolute values and percentages of predicted normal values.

#### Arterial blood gas analysis.

Arterial blood gas measurements were obtained during the same clinical encounter while patients were breathing room air (for non-LTOT patients) or their prescribed oxygen flow rate (for LTOT patients). Parameters included partial pressure of oxygen (PaO_2_), partial pressure of carbon dioxide (PaCO_2_), and oxygen saturation (SaO_2_).

#### Laboratory assessments.

Venous blood samples were collected in the morning after overnight fasting and analyzed in the central laboratory using standardized automated systems.

The neutrophil-to-lymphocyte ratio (NLR) was calculated as the ratio of absolute neutrophil count to absolute lymphocyte count.

### Statistical analysis

Statistical analyses were performed using SPSS software (version 24.0; IBM Corp., Armonk, NY, USA). Descriptive statistics included means ± standard deviations for continuous variables and frequencies with percentages for categorical variables.

Normality of continuous variables was assessed using the Kolmogorov-Smirnov test and visual inspection of histograms. Due to non-normal distribution of NLR, square-root log transformation (SqrtLogNLR) was applied to achieve normal distribution for correlation and regression analyses.

Comparisons between LTOT and non-LTOT groups were performed using the Mann-Whitney U test for continuous variables due to non-normal distribution of most variables. Chi-square or Fisher's exact tests were used for categorical variables as appropriate.

Correlation analyses were performed using Pearson correlation coefficients after appropriate transformation. To avoid mathematical coupling, neutrophil and lymphocyte counts were excluded from correlation analyses with NLR.

Variables showing significant associations in univariate analysis (p < 0.05) were entered into a multivariable linear regression model with SqrtLogNLR as the dependent variable. Variance inflation factors (VIF) were calculated to assess multicollinearity, with VIF > 5 considered indicative of significant multicollinearity.

A two-tailed p-value < 0.05 was considered statistically significant for all analyses.

Given the exploratory nature of this secondary analysis, no formal sample size calculation was performed. The analysis included all patients meeting inclusion criteria from the original prospective cohort. Results should be interpreted as hypothesis-generating rather than confirmatory. Furthermore, the multivariable regression model was intended to explore independent associations with NLR rather than to develop a predictive model. Variables were selected based on significant univariate associations and biological plausibility. Therefore, the findings of the multivariable analysis should be interpreted cautiously and require validation in larger independent cohorts.

## Results

### Baseline characteristics

A total of 127 clinically stable COPD patients were included in the analysis. Of these, 31 patients (24.4%) were receiving long-term oxygen therapy (LTOT), while 96 patients (75.6%) were not. The baseline demographic, clinical, and laboratory characteristics of the study population are presented in Table 1.

**Table 1:** Baseline characteristics of the study population.

Variable	COPD Patients (n = 127)
Demographic and Clinical Characteristics	
Age (years)	67 ± 9
Sex (female/male), n	12/115
Body mass index (kg/m^2^)	26 ± 5
Current smoking (yes/no), n	22/105
Smoking history (pack-years)	34 ± 18
Duration of bronchodilator therapy (years)	7 ± 7
Long-term oxygen therapy (yes/no), n	31/96
Inflammatory and Hematological Parameters	
White blood cell count (×10^3^/μL)	8.66 ± 2.7
Neutrophils (×10^3^/μL)	6.12 ± 2.6
Lymphocytes (×10^3^/μL)	1.8 ± 0.8
Eosinophils (×10^3^/μL)	0.17 ± 0.16
Neutrophil-to-lymphocyte ratio	4.33 ± 3.4
C-reactive protein (mg/L)	12 ± 13
Pulmonary Function and Gas Exchange	
FEV_1_ (% predicted)	52 ± 16
FVC (% predicted)	70 ± 18
FEV_1_/FVC ratio (%)	56 ± 8
PaO_2_ (mmHg)	61 ± 13
PaCO_2_ (mmHg)	43 ± 13
Oxygen saturation (%)	92 ± 4

Data are presented as mean ± standard deviation for continuous variables and as number (n) for categorical variables.

Abbreviations: COPD, chronic obstructive pulmonary disease; FEV_1_, forced expiratory volume in one second; FVC, forced vital capacity; PaO_2_, partial pressure of arterial oxygen; PaCO_2_, partial pressure of arterial carbon dioxide.

### Demographic and clinical characteristics

The study population was predominantly male (90.6%) with a mean age of 67 ± 9 years. Most patients were current or former smokers, with a mean smoking history of 34 ± 18 pack-years. The mean duration of bronchodilator therapy was 7 ± 7 years, reflecting the chronic nature of the disease in this cohort.

### Inflammatory and hematological parameters

The mean neutrophil-to-lymphocyte ratio for the entire cohort was 4.33 ± 3.4. Mean total white blood cell count was 8.66 ± 2.7 × 10^3^/μL, with neutrophil count of 6.12 ± 2.6 × 10^3^/μL and lymphocyte count of 1.8 ± 0.8 × 10^3^/μL. Eosinophil count was 0.17 ± 0.16 × 10^3^/μL, and mean C-reactive protein level was 12 ± 13 mg/L.

### Pulmonary function and gas exchange parameters

The overall study population demonstrated moderate to severe airflow limitation, with mean post-bronchodilator FEV_1_ of 52 ± 16% predicted and FEV_1_/FVC ratio of 56 ± 8%. Mean arterial oxygen tension was 61 ± 13 mmHg, with oxygen saturation of 92 ± 4%, reflecting the presence of patients with varying degrees of hypoxemia.

### Comparison according to LTOT status

Patients receiving LTOT demonstrated significantly more advanced disease compared to those not receiving oxygen therapy (Table 2). LTOT-treated patients had longer duration of bronchodilator therapy (10 ± 7 vs 6 ± 7 years, p = 0.001), indicating longer disease duration.

**Table 2:** Comparison of clinical and laboratory parameters according to LTOT status.

Variable	LTOT Group (n = 31)	Non-LTOT Group (n = 96)	p-value
Duration of bronchodilator therapy (years)	10 ± 7	6 ± 7	0.001*
PaO_2_ (mmHg)	58 ± 14	63 ± 12	0.159
PaCO_2_ (mmHg)	51 ± 13	36 ± 5	<0.001*
Oxygen saturation (%)	88 ± 4	94 ± 3	<0.001*
FEV_1_ (L)	1.06 ± 0.42	1.52 ± 0.43	<0.001*
FVC (L)	1.97 ± 0.63	2.62 ± 0.67	<0.001*
FEV_1_/FVC ratio (%)	53 ± 8	58 ± 7	0.006*
White blood cell count (×10^3^/μL)	8.95 ± 2.85	8.57 ± 2.73	0.519
Neutrophils (×10^3^/μL)	6.69 ± 2.82	5.94 ± 2.45	0.214
Lymphocytes (×10^3^/μL)	1.41 ± 0.68	1.87 ± 0.72	0.002*
Eosinophils (cells/μL)	113 ± 95	182 ± 178	0.049*
C-reactive protein (mg/L)	11 ± 9	13 ± 14	0.950
Neutrophil-to-Lymphocyte Ratio	6.32 ± 4.87	3.68 ± 2.41	0.001 *

Data are presented as mean ± standard deviation for continuous variables. Between-group comparisons were performed using Mann-Whitney U test for continuous variables and chi-square test for categorical variables. Two-tailed p-values are reported.

*Statistically significant (p < 0.05)

Abbreviations: LTOT, long-term oxygen therapy; PaO_2_, partial pressure of arterial oxygen; PaCO_2_, partial pressure of arterial carbon dioxide; FEV_1_, forced expiratory volume in one second; FVC, forced vital capacity.

Pulmonary function parameters were significantly worse in the LTOT group, with lower FEV_1_ (1.06 ± 0.42 vs 1.52 ± 0.43 L, p < 0.001) and FVC (1.97 ± 0.63 vs 2.62 ± 0.67 L, p < 0.001). The FEV1/FVC ratio was also significantly reduced in LTOT patients (53 ± 8 vs 58 ± 7%, p = 0.006).

Despite the more advanced disease profile in LTOT patients, conventional inflammatory markers showed no significant differences between groups. Total white blood cell count was similar between LTOT and non-LTOT patients (8.95 ± 2.85 vs 8.57 ± 2.73 × 10^3^/μL, p = 0.519). C-reactive protein levels were also comparable (11 ± 9 vs 13 ± 14 mg/L, p = 0.950).

In contrast, significant differences were observed in immune cell distribution. LTOT patients had significantly lower lymphocyte counts (1.41 ± 0.68 vs 1.87 ± 0.72 × 10^3^/μL, p = 0.002) and lower eosinophil counts (113 ± 95 vs 182 ± 178 cells/μL, p = 0.049). Neutrophil counts showed a trend toward higher values in the LTOT group, though this difference did not reach statistical significance (6.69 ± 2.82 vs 5.94 ± 2.45 × 10³/μL, p = 0.214).

### Neutrophil-to-lymphocyte ratio

The most striking finding was the significantly elevated NLR in LTOT patients compared to non-LTOT patients (6.32 ± 4.87 vs 3.68 ± 2.41, p = 0.001). This elevation occurred despite the absence of differences in conventional inflammatory markers, suggesting that the increased NLR was primarily driven by lymphopenia rather than neutrophilia or overall inflammatory activation.

### Correlation analysis

Correlation analyses were performed using square-root log-transformed NLR (SqrtLogNLR) to account for non-normal distribution. The results revealed several significant associations (Table 3).

**Table 3:** Correlation analysis between SqrtLogNLR and selected variables.

Variable	r	p-value
FEV_1_ (% predicted)	−0.215	0.015*
FVC (% predicted)	−0.229	0.010*
FEV_1_ (L)	−0.256	0.004*
FVC (L)	−0.265	0.003*
PaO_2_ (mmHg)	−0.115	0.205
PaCO_2_ (mmHg)	0.123	0.495
Oxygen saturation (%)	−0.252	0.004*
White blood cell count (×10^3^/μL)	0.441	<0.001*
Eosinophils (cells/μL)	−0.442	<0.001*
C-reactive protein (mg/L)	0.275	0.014*
Albumin (g/dL)	0.385	0.002*
Lactate dehydrogenase (U/L)	0.322	0.018*

Correlation analyses were performed using Pearson correlation analysis between square-root log-transformed neutrophil-to-lymphocyte ratio (SqrtLogNLR) and selected continuous variables. Data transformation was applied to achieve normal distribution. Two-tailed p-values are reported. Neutrophil and lymphocyte counts were excluded from analysis to avoid mathematical coupling with NLR.

*Statistically significant (p < 0.05)

Abbreviations: SqrtLogNLR, square-root log-transformed neutrophil-to-lymphocyte ratio; r, Pearson correlation coefficient; FEV_1_, forced expiratory volume in one second; FVC, forced vital capacity; PaO_2_, partial pressure of arterial oxygen; PaCO_2_, partial pressure of arterial carbon dioxide.

SqrtLogNLR showed a moderate positive correlation with total white blood cell count (r = 0.441, p < 0.001) and C-reactive protein (r = 0.275, p = 0.014), suggesting partial alignment between NLR and conventional inflammatory markers. However, a strong negative correlation was observed with eosinophil count (r = −0.442, p < 0.001).

Weak to moderate negative correlations were identified between SqrtLogNLR and pulmonary function parameters, including FEV_1_ % predicted (r = −0.215, p = 0.015), FVC % predicted (r = −0.229, p = 0.010), and absolute values of FEV_1_ (r = −0.256, p = 0.004) and FVC (r = −0.265, p = 0.003).

Gas exchange parameters also showed significant associations, with a negative correlation between SqrtLogNLR and oxygen saturation (r = −0.252, p = 0.004). However, no significant correlation was found with PaO_2_ (r = −0.227, p = 0.205) or PaCO_2_ (r = 0.123, p = 0.495).

### Multivariable analysis

Variables that demonstrated significant associations in univariate analysis were entered into a multivariable linear regression model to identify independent predictors of NLR elevation (Table 4).

**Table 4:** Multivariable linear regression analysis for SqrtLogNLR.

Variable	B	SE	β	t	p-value	95% CI	VIF
Constant	0.937	0.412	—	2.275	0.026	0.114–1.760	—
White blood cell count (×10³/μL)	0.022	0.006	0.362	3.947	<0.001*	0.011–0.033	1.095
C-reactive protein (mg/L)	0.003	0.001	0.189	2.105	0.039*	0.000–0.005	1.046
Eosinophils (cells/μL)	−0.0004	0.0001	−0.354	−3.986	<0.001*	−0.001–0.000	1.029
Oxygen saturation (%)	−0.005	0.005	−0.106	−1.033	0.305	−0.014–0.004	1.371
FVC (% predicted)	−0.0005	0.001	−0.050	−0.491	0.625	−0.002–0.001	1.367

*Multivariable linear regression analysis with square-root log-transformed neutrophil-to-lymphocyte ratio (SqrtLogNLR) as the dependent variable. Variables showing significant associations in univariate analysis (p < 0.05) were entered into the model. Model R² = 0.385, adjusted R² = 0.360, F = 15.124, p < 0.001.*

*Statistically significant (p < 0.05)

*Abbreviations: B, unstandardized regression coefficient; SE, standard error; β, standardized regression coefficient; CI, confidence interval; VIF, variance inflation factor; FVC, forced vital capacity.*

In the adjusted model, total white blood cell count (β = 0.362, p < 0.001), eosinophil count (β = −0.354, p < 0.001), and C-reactive protein (β = 0.189, p = 0.039) remained independently associated with SqrtLogNLR. These associations suggest that NLR elevation is partially related to conventional inflammatory markers and eosinophil depletion.

Importantly, neither oxygenation parameters (oxygen saturation: β = −0.106, p = 0.305) nor pulmonary function measures (FVC: β = −0.050, p = 0.625) maintained independent associations with NLR in the multivariable model. This finding suggests that the relationship between NLR and disease severity may be mediated through inflammatory pathways rather than direct physiological impairment.

Variance inflation factors were below 2 for all variables, indicating absence of significant multicollinearity.

## Discussion

Most previous studies evaluating NLR in COPD have focused on its association with disease severity, exacerbation risk, and mortality, generally interpreting elevated NLR as a surrogate marker of increased systemic inflammation. In contrast, the present study was not designed to re-examine these established associations. Rather, it sought to determine what elevated NLR biologically represents in patients with advanced hypoxemic COPD receiving long-term oxygen therapy.

This study demonstrates a distinct inflammatory phenotype in clinically stable COPD patients with chronic hypoxemia, characterized by elevated neutrophil-to-lymphocyte ratio (NLR) occurring independently of conventional inflammatory markers. The key finding is that patients requiring long-term oxygen therapy exhibit significantly higher NLR values despite comparable levels of C-reactive protein and total white blood cell count, suggesting that NLR elevation in advanced COPD may reflect immune cell redistribution rather than classical systemic inflammatory activation.

The concept of “inflammatory discordance” emerges as a central finding of this study. In most clinical contexts, elevated NLR is interpreted as a marker of increased systemic inflammation, typically accompanied by elevations in acute-phase reactants such as CRP and increased leukocyte counts [9,17]. Our findings challenge this assumption in the context of advanced COPD, where NLR elevation occurs without corresponding changes in conventional inflammatory markers.

This discordance suggests that NLR may serve dual roles in COPD: as a traditional inflammatory marker in some contexts and as an indicator of altered immune cell distribution in others. The biological significance of this distinction is substantial, as it implies that similar NLR values may represent fundamentally different pathophysiological states depending on the clinical setting [14,18].

Several mechanisms may explain the observed dissociation between NLR and conventional inflammatory markers in LTOT-treated patients. Chronic hypoxemia has been shown to influence immune cell trafficking and survival through hypoxia-inducible factor (HIF) pathways [19,20]. Hypoxic stress can promote neutrophil survival and activation while simultaneously inducing lymphocyte apoptosis, leading to altered circulating cell ratios without necessarily triggering an acute-phase response [21].

Furthermore, chronic hypercapnia, which was significantly elevated in our LTOT group, has immunomodulatory effects that may contribute to lymphopenia through direct effects on T-cell function and survival [22]. The neuroendocrine stress response associated with chronic respiratory failure may also influence immune cell distribution through cortisol-mediated mechanisms, promoting lymphocyte sequestration or apoptosis [23].

Oxidative stress, a hallmark of advanced COPD, represents another potential mechanism. Chronic exposure to reactive oxygen species can induce lymphocyte dysfunction and death while promoting neutrophil activation and survival, contributing to the observed cellular imbalance [24]. Importantly, these mechanisms may operate independently of classical inflammatory pathways, explaining the absence of corresponding CRP elevation.

Our findings have important implications for the clinical interpretation of NLR in COPD patients. The traditional approach of viewing elevated NLR solely as an indicator of systemic inflammation may be insufficient in advanced disease. Instead, clinicians should consider the broader inflammatory context, including conventional markers such as CRP and total leukocyte count, when interpreting NLR values.

This context-dependent interpretation is particularly relevant for therapeutic decision-making. If NLR elevation reflects immune cell redistribution rather than active inflammation, anti-inflammatory therapies targeting classical inflammatory pathways may be less effective in these patients. Alternative therapeutic approaches addressing specific aspects of immune dysfunction, such as lymphopenia or oxidative stress, might be more appropriate [25,26].

These findings contribute to the growing understanding of phenotypic heterogeneity in COPD. The “inflammatory discordance” phenotype identified in this study represents patients with advanced disease who may not respond optimally to broad anti-inflammatory strategies.

This phenotypic classification has potential implications for personalized medicine approaches in COPD. Stratifying patients based on their inflammatory profile, rather than relying solely on traditional severity measures, may improve treatment selection and clinical outcomes [27,28].

The observation that NLR elevation in LTOT patients occurs independently of conventional inflammatory markers raises questions about its prognostic significance. Previous studies have consistently shown associations between elevated NLR and adverse outcomes in COPD, including increased mortality and exacerbation risk [10–13]. However, our findings suggest that the mechanisms underlying these associations may differ between patient subgroups.

In patients with classical inflammatory activation, NLR elevation likely reflects increased systemic inflammatory burden with its associated adverse effects on cardiovascular and other organ systems. In contrast, in patients with the inflammatory discordance phenotype, NLR elevation may primarily reflect immune dysfunction and impaired host defense mechanisms, potentially leading to increased infection risk rather than inflammatory complications [29].

The identification of this distinct inflammatory phenotype has several therapeutic implications. First, it suggests that the “one-size-fits-all” approach to anti-inflammatory therapy in COPD may be inadequate. Patients with elevated NLR but normal conventional inflammatory markers may require interventions targeting specific aspects of immune dysfunction rather than broad inflammatory suppression.

Second, these findings highlight the potential importance of addressing the underlying causes of immune cell redistribution, such as chronic hypoxemia and oxidative stress. Optimization of oxygen therapy, pulmonary rehabilitation, and antioxidant strategies may be particularly beneficial in this patient subgroup [30,31].

Finally, the results suggest that clinical trials evaluating anti-inflammatory therapies in COPD should stratify patients by inflammatory phenotype. This approach may help identify subgroups most likely to benefit from specific interventions and explain some of the heterogeneous results observed in previous studies [32].

Several limitations should be acknowledged. The cross-sectional design prevents assessment of causality or temporal relationships between inflammatory markers and disease progression. The single-center design and relatively small sample size, particularly in the LTOT subgroup, may limit generalizability of the findings.

In addition, the relatively limited number of LTOT-treated patients should be considered when interpreting the multivariable regression analysis. Although the model was designed as an exploratory analysis using clinically relevant variables selected on the basis of biological plausibility and univariate associations, confirmation of these findings in larger independent cohorts would strengthen confidence in the observed associations.

The study was limited to conventional inflammatory markers and did not include more sophisticated immunological assessments such as cytokine profiling or immune cell subset analysis. Such detailed analyses might provide additional insights into the mechanisms underlying the observed inflammatory discordance.

Furthermore, while LTOT status serves as a clinically relevant indicator of advanced disease with chronic hypoxemia, it may not capture the full spectrum of physiological abnormalities in advanced COPD. Some patients with severe disease may not meet current LTOT criteria despite having significant physiological impairment.

Medication effects, including inhaled and systemic corticosteroids, were not systematically evaluated and may have influenced inflammatory profiles. However, all patients were clinically stable at the time of assessment, reducing the likelihood of acute medication effects on inflammatory markers.

Several areas warrant further investigation. Longitudinal studies are needed to determine whether the inflammatory discordance phenotype predicts specific clinical outcomes such as infection rates, exacerbation patterns, or mortality. Additionally, more detailed immunological analyses, including assessment of immune cell subpopulations, cytokine profiles, and functional immune responses, could provide insights into the mechanisms underlying this phenotype.

The relationship between therapeutic interventions and inflammatory phenotypes also requires exploration. Specifically, it would be valuable to determine whether anti-inflammatory treatments have differential effects in patients with concordant versus discordant inflammatory profiles.

## Conclusions

This study identifies a distinct inflammatory phenotype in advanced COPD characterized by elevated NLR occurring independently of conventional inflammatory markers. The findings suggest that NLR elevation in LTOT-treated patients reflects immune cell redistribution rather than classical systemic inflammatory activation, highlighting the importance of context-dependent biomarker interpretation in COPD.

These results contribute to the understanding of phenotypic heterogeneity in COPD and have important implications for biomarker interpretation, therapeutic decision-making, and clinical trial design. The identification of the inflammatory discordance phenotype supports the move toward personalized medicine approaches in COPD management and suggests that therapeutic strategies should be tailored to specific inflammatory profiles rather than applying uniform anti-inflammatory approaches to all patients.
