# Supplementary material for: Inflammatory discordance phenotype in COPD: Dissociation between neutrophil-to-lymphocyte ratio and conventional markers
Source: PLoS One. 2026 Sep 11;21(9):e0357923. doi: 10.1371/journal.pone.0357923 (PMC13567818; doi:10.1371/journal.pone.0357923)
Supplement: Supplementary file 2 [file pone.0357923.s004.docx]

Manuscript Title:

Inflammatory Discordance Phenotype in COPD: Dissociation Between Neutrophil-to-Lymphocyte Ratio and Conventional Markers

Manuscript ID:

PONE-D-26-20604

We would like to thank the Academic Editor and the Reviewers for their careful evaluation of our manuscript and for their constructive comments. We have revised the manuscript accordingly and believe that these revisions have substantially strengthened the paper. Detailed responses to each comment are provided below.

ACADEMIC EDITOR

Comment 1:

NOVELTY AND POSITIONING IN THE LITERATURE

Response:

We thank the Editor for this valuable comment. To better position our findings within the existing literature, we revised the Discussion section to clarify that the principal contribution of the study is not the previously established association between NLR and COPD severity, but the observation of a dissociation between NLR and conventional inflammatory markers in LTOT-treated patients. We further clarified how this observation differs from previous studies that primarily evaluated NLR as a marker of disease severity, exacerbation risk, or mortality. The revised Discussion now emphasizes that elevated NLR in advanced hypoxemic COPD may not uniformly reflect increased systemic inflammatory burden and may instead represent a distinct inflammatory phenotype characterized by discordance between NLR and conventional inflammatory markers.

Comment 2:

MULTIVARIABLE REGRESSION MODEL AND SAMPLE SIZE ADEQUACY

Response:

We thank the Editor for highlighting this important methodological consideration. The multivariable regression analysis was designed as an exploratory, hypothesis-generating analysis rather than a predictive model. Variables were selected based on significant univariate associations and biological plausibility. To address this concern, we added clarifying statements in both the Methods and Limitations sections regarding the exploratory nature of the analysis, the absence of a formal sample size calculation for this secondary analysis, and the need for validation of these findings in larger independent cohorts.

REVIEWER #1

Comment:

At present the findings appear largely confirmatory rather than hypothesis generating. There is so much literature available on current topic or very much similar topic. I do not see any novelty in this manuscript.

Response:

We thank the reviewer for this thoughtful comment. We agree that numerous previous studies have investigated the association between NLR and COPD severity, exacerbation risk, and mortality. However, the primary objective of the present study was not to re-confirm these established associations.

Rather, this study was designed to examine whether elevated NLR remains biologically coupled to conventional inflammatory markers in patients with advanced hypoxemic COPD receiving long-term oxygen therapy (LTOT). Our findings demonstrate that LTOT-treated patients exhibited significantly elevated NLR values despite comparable CRP and total leukocyte levels, suggesting a dissociation between NLR and conventional markers of systemic inflammation.

To address the reviewer's concern, we further clarified the positioning of our findings within the existing literature and highlighted more explicitly that the principal contribution of the study is not the association between NLR and disease severity, which has been extensively reported previously, but the observation of a dissociation between NLR and conventional inflammatory markers in LTOT-treated patients. These findings suggest the presence of an inflammatory discordance phenotype in advanced hypoxemic COPD and provide a different biological interpretation of elevated NLR in this specific clinical context.

REVIEWER #2

Comment:

With only 31 patients in the LTOT group, the multivariable regression model includes 5 predictor variables. This may exceed appropriate variable-to-event ratios for regression modeling and risk overfitting. The authors should comment on the adequacy of the sample size for the multivariable model, or consider limiting the number of predictors.

Response:

We thank the reviewer for this important comment. We acknowledge that the relatively small number of LTOT-treated patients should be considered when interpreting the multivariable regression analysis.

The regression model was designed as an exploratory, hypothesis-generating analysis rather than a predictive model. Variables entered into the multivariable model were selected based on significant univariate associations and biological plausibility. Because the primary objective was to explore independent associations with NLR rather than to develop a prediction tool, we elected to retain the original model structure.

To address the reviewer's concern, we added clarifying statements in both the Methods and Limitations sections. These revisions explicitly state that no formal sample size calculation was performed for this secondary exploratory analysis and that confirmation in larger independent cohorts would strengthen confidence in the observed associations.

Additional Editorial Requirements

Response:

The Data Availability Statement was updated. The corresponding author was removed as the data access contact and replaced with a non-author institutional contact, the Sivas Cumhuriyet University Faculty of Medicine Health Sciences Research Ethics Committee, including updated contact information.

The author information in the submission system was amended to include Sefa Levent Özşahin, as requested.

The ethics statement was retained only within the Methods section. Any duplicate ethics approval statements appearing elsewhere in the manuscript were removed.

We appreciate the time and effort invested by the Academic Editor and the Reviewers in evaluating our manuscript. Their comments and suggestions have been invaluable in strengthening the work.

Sincerely,

Can Çolakoğlu, MD

Corresponding Author

On behalf of all authors
