# Supplementary material for: Inflammatory discordance phenotype in COPD: Dissociation between neutrophil-to-lymphocyte ratio and conventional markers
Source: PLoS One. 2026 Sep 11;21(9):e0357923. doi: 10.1371/journal.pone.0357923 (PMC13567818; doi:10.1371/journal.pone.0357923)
Supplement: Supplementary file 3 [file pone.0357923.s003.docx]

The revised manuscript is potentially suitable for publication. The authors have adequately clarified the exploratory nature and clinical positioning of the study. However, two minor but important issues should be addressed before acceptance.

1. The response concerning the sample size of the multivariable regression may create the impression that the model was based only on the 31 patients receiving LTOT. According to the manuscript, the linear regression appears to have been performed in the entire cohort of 127 patients. This should be stated explicitly in the Methods, Results, and response letter, together with the actual number of patients included in the complete-case regression analysis. Because this is a linear regression with a continuous dependent variable rather than an event-based model, the discussion should focus on the number of observations per predictor rather than a variable-to-event ratio.
2. The statement that NLR elevation occurred “independently of conventional inflammatory markers” is not fully consistent with the multivariable results. Both WBC and CRP remained independently associated with transformed NLR (WBC: β = 0.362, p < 0.001; CRP: β = 0.189, p = 0.039). The authors should therefore revise this statement throughout the Abstract, Discussion, and Conclusions. A more accurate interpretation would be that LTOT-treated patients exhibited higher NLR despite no statistically significant between-group differences in mean WBC or CRP levels, while NLR nevertheless retained partial associations with these conventional inflammatory markers in the overall cohort.

Subject to these clarifications and correction of the overstated interpretation, the manuscript would be suitable for publication.

Recommendation: Minor revision.
