# Supplementary material for: Inflammatory discordance phenotype in COPD: Dissociation between neutrophil-to-lymphocyte ratio and conventional markers
Source: PLoS One. 2026 Sep 11;21(9):e0357923. doi: 10.1371/journal.pone.0357923 (PMC13567818; doi:10.1371/journal.pone.0357923)
Supplement: Peer review history

# Peer review: Decision Letter 0

**DOI:** 10.1371/journal.pone.0357923.r001

**Authors:** Jamal Akhtar

27 May 2026

Dear Dr. colakoglu,

Thank you for submitting your manuscript to PLOS ONE. After careful consideration, we feel that it has merit but does not fully meet PLOS ONE’s publication criteria as it currently stands. Therefore, we invite you to submit a revised version of the manuscript that addresses the points raised during the review process.

Please submit your revised manuscript by Jul 26 2026 11:59PM. If you will need more time than this to complete your revisions, please reply to this message or contact the journal office at plosone@plos.org. When you're ready to submit your revision, log on to https://www.editorialmanager.com/pone/ and select the 'Submissions Needing Revision' folder to locate your manuscript file.

- A letter that responds to each point raised by the academic editor and reviewer(s). You should upload this letter as a separate file labeled 'Response to Reviewers'.
- A marked-up copy of your manuscript that highlights changes made to the original version. You should upload this as a separate file labeled 'Revised Manuscript with Track Changes'.
- An unmarked version of your revised paper without tracked changes. You should upload this as a separate file labeled 'Manuscript'.

If you would like to make changes to your financial disclosure, please include your updated statement in your cover letter. Guidelines for resubmitting your figure files are available below the reviewer comments at the end of this letter.

If applicable, we recommend that you deposit your laboratory protocols in protocols.io to enhance the reproducibility of your results. Protocols.io assigns your protocol its own identifier (DOI) so that it can be cited independently in the future. For instructions see: https://journals.plos.org/plosone/s/submission-guidelines#loc-laboratory-protocols. Additionally, PLOS ONE offers an option for publishing peer-reviewed Lab Protocol articles, which describe protocols hosted on protocols.io. Read more information on sharing protocols at https://plos.org/protocols?utm_medium=editorial-email&utm_source=authorletters&utm_campaign=protocols.

As the corresponding author, your ORCID iD is verified in the submission system and will appear in the published article. PLOS supports the use of ORCID, and we encourage all coauthors to register for an ORCID iD and use it as well. Please encourage your coauthors to verify their ORCID iD within the submission system before final acceptance, as unverified ORCID iDs will not appear in the published article. *Only*  the individual author can complete the verification step; PLOS staff *cannot*  verify ORCID iDs on behalf of authors.

We look forward to receiving your revised manuscript.

Kind regards,

Jamal Akhtar

Academic Editor

PLOS One

Journal Requirements:

When submitting your revision, we need you to address these additional requirements.

1. Please ensure that your manuscript meets PLOS ONE's style requirements, including those for file naming. The PLOS ONE style templates can be found at

https://journals.plos.org/plosone/s/file?id=wjVg/PLOSOne_formatting_sample_main_body.pdf and

https://journals.plos.org/plosone/s/file?id=ba62/PLOSOne_formatting_sample_title_authors_affiliations.pdf.

2. In the online submission form you indicate that your data is not available for proprietary reasons and have provided a contact point for accessing this data. Please note that your current contact point is a co-author on this manuscript. According to our Data Policy, the contact point must not be an author on the manuscript and must be an institutional contact, ideally not an individual. Please revise your data statement to a non-author institutional point of contact, such as a data access or ethics committee, and send this to us via return email. Please also include contact information for the third party organization, and please include the full citation of where the data can be found.

3. Please amend the manuscript submission data (via Edit Submission) to include author Sefa Levent Özşahin.

4. Your ethics statement should only appear in the Methods section of your manuscript. If your ethics statement is written in any section besides the Methods, please delete it from any other section.

5. If the reviewer comments include a recommendation to cite specific previously published works, please review and evaluate these publications to determine whether they are relevant and should be cited. There is no requirement to cite these works unless the editor has indicated otherwise.

Additional Editor Comments (if provided):

Thank you for submitting your manuscript "Inflammatory Discordance Phenotype in COPD: Dissociation Between Neutrophil-to-Lymphocyte Ratio and Conventional Markers" (PONE-D-26-20604) to PLOS ONE. The manuscript has been reviewed by two expert reviewers whose comments are provided below. After careful evaluation, I am requesting a Major Revision before a final decision can be made.

The reviewers hold divergent views. One reviewer finds the concept of inflammatory discordance in COPD clinically meaningful, while the other raises concerns about novelty relative to the existing literature. I have reviewed both perspectives and determined that the manuscript addresses an important clinical question but requires substantive revision to address the following concerns:

1. NOVELTY AND POSITIONING IN THE LITERATURE (Reviewer 1 concern)

The manuscript must more clearly and explicitly articulate what is novel about the findings, specifically how the concept of "inflammatory discordance" between NLR and conventional markers (CRP, ESR, WBC) provides new insight beyond what is already established in the COPD inflammatory biomarker literature. A focused literature review paragraph in the Introduction and/or Discussion should demonstrate the gap this study fills. Simply confirming known associations is insufficient; the specific phenotypic characterization and clinical implications of the discordance must be highlighted.

2. MULTIVARIABLE REGRESSION MODEL AND SAMPLE SIZE ADEQUACY (Reviewer 2 concern)

The multivariable regression model for the LTOT subgroup includes 5 predictor variables with only 31 patients. This exceeds the commonly recommended minimum of 10 events per predictor variable and raises the risk of model overfitting. The authors should:

a) Comment explicitly on the sample size adequacy for the multivariable model in the Methods and/or Limitations section.

b) Consider reducing the number of predictors (e.g., by using a priori-defined variables based on clinical rationale) or applying penalized regression methods (e.g., ridge or LASSO regression).

c) Alternatively, provide a clear clinical and statistical justification for retaining the current model structure.

Please provide a point-by-point response to each reviewer comment in your revision. The manuscript has technical merit and addresses a clinically relevant phenotype in COPD; with appropriate revisions it may meet PLOS ONE's publication criteria.

We look forward to receiving your revised manuscript.

[Note: HTML markup is below. Please do not edit.]

Reviewers' comments:

Reviewer's Responses to Questions

**Comments to the Author**

1. Is the manuscript technically sound, and do the data support the conclusions?

Reviewer #1: Partly

Reviewer #2: Yes

**********

2. Has the statistical analysis been performed appropriately and rigorously?

Reviewer #1: Yes

Reviewer #2: Yes

**********

3. Have the authors made all data underlying the findings in their manuscript fully available?

The PLOS Data policy

Reviewer #1: Yes

Reviewer #2: Yes

**********

4. Is the manuscript presented in an intelligible fashion and written in standard English?

Reviewer #1: Yes

Reviewer #2: Yes

**********

Reviewer #1: At present the findings appear largely confirmatory rather than hypothesis generating. There is so much literature available on current topic or very much similar topic. i do not see any novelty in this manuscript.

Reviewer #2: This manuscript addresses a clinically relevant and conceptually interesting question. The study is clearly written, the statistical approach is generally appropriate, and the concept of "inflammatory discordance" is a meaningful contribution to COPD phenotyping literature. I recommend acceptance.

Comments:

With only 31 patients in the LTOT group, the multivariable regression model includes 5 predictor variables. This may exceed appropriate variable-to-event ratios for regression modeling and risk overfitting. The authors should comment on the adequacy of the sample size for the multivariable model, or consider limiting the number of predictors.

**********

what does this mean?). If published, this will include your full peer review and any attached files.

If you choose “no”, your identity will remain anonymous but your review may still be made public.

**Do you want your identity to be public for this peer review?**  For information about this choice, including consent withdrawal, please see our Privacy Policy

Reviewer #1: **Yes:**  Devendra Tripathi

Reviewer #2: No

**********

[NOTE: If reviewer comments were submitted as an attachment file, they will be attached to this email and accessible via the submission site. Please log into your account, locate the manuscript record, and check for the action link "View Attachments". If this link does not appear, there are no attachment files.]

To ensure your figures meet our technical requirements, please review our figure guidelines: https://journals.plos.org/plosone/s/figures

You may also use PLOS’s free figure tool, NAAS, to help you prepare publication quality figures: https://journals.plos.org/plosone/s/figures#loc-tools-for-figure-preparation.

NAAS will assess whether your figures meet our technical requirements by comparing each figure against our figure specifications.

Attachment Submitted filename: COPD, NLR -The topic is cli.textClipping

# Author comment: Author response to Decision Letter 1

**DOI:** 10.1371/journal.pone.0357923.r002

26 Jun 2026

Manuscript Title:

Inflammatory Discordance Phenotype in COPD: Dissociation Between Neutrophil-to-Lymphocyte Ratio and Conventional Markers

Manuscript ID:

PONE-D-26-20604

We would like to thank the Academic Editor and the Reviewers for their careful evaluation of our manuscript and for their constructive comments. We have revised the manuscript accordingly and believe that these revisions have substantially strengthened the paper. Detailed responses to each comment are provided below.

ACADEMIC EDITOR

Comment 1:

NOVELTY AND POSITIONING IN THE LITERATURE

Response:

We thank the Editor for this valuable comment. To better position our findings within the existing literature, we revised the Discussion section to clarify that the principal contribution of the study is not the previously established association between NLR and COPD severity, but the observation of a dissociation between NLR and conventional inflammatory markers in LTOT-treated patients. We further clarified how this observation differs from previous studies that primarily evaluated NLR as a marker of disease severity, exacerbation risk, or mortality. The revised Discussion now emphasizes that elevated NLR in advanced hypoxemic COPD may not uniformly reflect increased systemic inflammatory burden and may instead represent a distinct inflammatory phenotype characterized by discordance between NLR and conventional inflammatory markers.

Comment 2:

MULTIVARIABLE REGRESSION MODEL AND SAMPLE SIZE ADEQUACY

Response:

We thank the Editor for highlighting this important methodological consideration. The multivariable regression analysis was designed as an exploratory, hypothesis-generating analysis rather than a predictive model. Variables were selected based on significant univariate associations and biological plausibility. To address this concern, we added clarifying statements in both the Methods and Limitations sections regarding the exploratory nature of the analysis, the absence of a formal sample size calculation for this secondary analysis, and the need for validation of these findings in larger independent cohorts.

REVIEWER #1

Comment:

At present the findings appear largely confirmatory rather than hypothesis generating. There is so much literature available on current topic or very much similar topic. I do not see any novelty in this manuscript.

Response:

We thank the reviewer for this thoughtful comment. We agree that numerous previous studies have investigated the association between NLR and COPD severity, exacerbation risk, and mortality. However, the primary objective of the present study was not to re-confirm these established associations.

Rather, this study was designed to examine whether elevated NLR remains biologically coupled to conventional inflammatory markers in patients with advanced hypoxemic COPD receiving long-term oxygen therapy (LTOT). Our findings demonstrate that LTOT-treated patients exhibited significantly elevated NLR values despite comparable CRP and total leukocyte levels, suggesting a dissociation between NLR and conventional markers of systemic inflammation.

To address the reviewer's concern, we further clarified the positioning of our findings within the existing literature and highlighted more explicitly that the principal contribution of the study is not the association between NLR and disease severity, which has been extensively reported previously, but the observation of a dissociation between NLR and conventional inflammatory markers in LTOT-treated patients. These findings suggest the presence of an inflammatory discordance phenotype in advanced hypoxemic COPD and provide a different biological interpretation of elevated NLR in this specific clinical context.

REVIEWER #2

Comment:

With only 31 patients in the LTOT group, the multivariable regression model includes 5 predictor variables. This may exceed appropriate variable-to-event ratios for regression modeling and risk overfitting. The authors should comment on the adequacy of the sample size for the multivariable model, or consider limiting the number of predictors.

Response:

We thank the reviewer for this important comment. We acknowledge that the relatively small number of LTOT-treated patients should be considered when interpreting the multivariable regression analysis.

The regression model was designed as an exploratory, hypothesis-generating analysis rather than a predictive model. Variables entered into the multivariable model were selected based on significant univariate associations and biological plausibility. Because the primary objective was to explore independent associations with NLR rather than to develop a prediction tool, we elected to retain the original model structure.

To address the reviewer's concern, we added clarifying statements in both the Methods and Limitations sections. These revisions explicitly state that no formal sample size calculation was performed for this secondary exploratory analysis and that confirmation in larger independent cohorts would strengthen confidence in the observed associations.

Additional Editorial Requirements

Response:

The Data Availability Statement was updated. The corresponding author was removed as the data access contact and replaced with a non-author institutional contact, the Sivas Cumhuriyet University Faculty of Medicine Health Sciences Research Ethics Committee, including updated contact information.

The author information in the submission system was amended to include Sefa Levent Özşahin, as requested.

The ethics statement was retained only within the Methods section. Any duplicate ethics approval statements appearing elsewhere in the manuscript were removed.

We appreciate the time and effort invested by the Academic Editor and the Reviewers in evaluating our manuscript. Their comments and suggestions have been invaluable in strengthening the work.

Sincerely,

Can Çolakoğlu, MD

Corresponding Author

On behalf of all authors

Attachment Submitted filename: Response to Reviewers.docx

# Peer review: Decision Letter 1

**DOI:** 10.1371/journal.pone.0357923.r003

**Authors:** Chiara Lazzeri

24 Aug 2026

Inflammatory Discordance Phenotype in COPD: Dissociation Between Neutrophil-to-Lymphocyte Ratio and Conventional Markers

PONE-D-26-20604R1

Dear Dr. colakoglu,

We’re pleased to inform you that your manuscript has been judged scientifically suitable for publication and will be formally accepted for publication once it meets all outstanding technical requirements.

Within one week, you’ll receive an e-mail detailing the required amendments. When these have been addressed, you’ll receive a formal acceptance letter and your manuscript will be scheduled for publication.

An invoice will be generated when your article is formally accepted. Please note, if your institution has a publishing partnership with PLOS and your article meets the relevant criteria, all or part of your publication costs will be covered. Please make sure your user information is up-to-date by logging into Editorial Manager at Editorial Manager® and clicking the ‘Update My Information' link at the top of the page. For questions related to billing, please contact billing support.

If your institution or institutions have a press office, please notify them about your upcoming paper to help maximize its impact. If they’ll be preparing press materials, please inform our press team as soon as possible -- no later than 48 hours after receiving the formal acceptance. Your manuscript will remain under strict press embargo until 2 pm Eastern Time on the date of publication. For more information, please contact onepress@plos.org.

Kind regards,

Chiara Lazzeri

Academic Editor

PLOS One

Additional Editor Comments (optional):

Reviewers' comments:

Reviewer's Responses to Questions

**Comments to the Author**

Reviewer #1: All comments have been addressed

Reviewer #3: (No Response)

**********

2. Is the manuscript technically sound, and do the data support the conclusions?

Reviewer #1: Yes

Reviewer #3: Yes

**********

3. Has the statistical analysis been performed appropriately and rigorously?

Reviewer #1: Yes

Reviewer #3: Yes

**********

4. Have the authors made all data underlying the findings in their manuscript fully available?

The PLOS Data policy

Reviewer #1: Yes

Reviewer #3: Yes

**********

5. Is the manuscript presented in an intelligible fashion and written in standard English?

Reviewer #1: Yes

Reviewer #3: Yes

**********

Reviewer #1: thank you for addressing all the comments and making requested revisions. The manuscript has been substantially improved and i am satisfied with writers response. and i have no further concerns.I recommend acceptance.

Reviewer #3: (No Response)

**********

what does this mean?). If published, this will include your full peer review and any attached files.

If you choose “no”, your identity will remain anonymous but your review may still be made public.

**Do you want your identity to be public for this peer review?**  For information about this choice, including consent withdrawal, please see our Privacy Policy

Reviewer #1: No

Reviewer #3: No

**********

Attachment Submitted filename: Review.docx

# Editor report: Acceptance letter

**DOI:** 10.1371/journal.pone.0357923.r004

**Authors:** Chiara Lazzeri

PONE-D-26-20604R1

PLOS One

Dear Dr. colakoglu,

I'm pleased to inform you that your manuscript has been deemed suitable for publication in PLOS One. Congratulations! Your manuscript is now being handed over to our production team.

At this stage, our production department will prepare your paper for publication. This includes ensuring the following:

* All references, tables, and figures are properly cited

* All relevant supporting information is included in the manuscript submission,

* There are no issues that prevent the paper from being properly typeset

You will receive further instructions from the production team, including instructions on how to review your proof when it is ready. Please keep in mind that we are working through a large volume of accepted articles, so please give us a few days to review your paper and let you know the next and final steps.

Lastly, if your institution or institutions have a press office, please let them know about your upcoming paper now to help maximize its impact. If they'll be preparing press materials, please inform our press team within the next 48 hours. Your manuscript will remain under strict press embargo until 2 pm Eastern Time on the date of publication. For more information, please contact onepress@plos.org.

You will receive an invoice from PLOS for your publication fee after your manuscript has reached the completed accept phase. If you receive an email requesting payment before acceptance or for any other service, this may be a phishing scheme. Learn how to identify phishing emails and protect your accounts at https://explore.plos.org/phishing.

If we can help with anything else, please email us at customercare@plos.org.

Thank you for submitting your work to PLOS One and supporting open access.

Kind regards,

PLOS One Editorial Office Staff

on behalf of

Dr. Chiara Lazzeri

Academic Editor

PLOS One
